# COVID-19 in Children: Expressions of Type I/II/III Interferons, TRIM28, SETDB1, and Endogenous Retroviruses in Mild and Severe Cases

**DOI:** 10.3390/ijms22147481

**Published:** 2021-07-13

**Authors:** Pier-Angelo Tovo, Silvia Garazzino, Valentina Daprà, Giulia Pruccoli, Cristina Calvi, Federica Mignone, Carla Alliaudi, Marco Denina, Carlo Scolfaro, Marisa Zoppo, Francesco Licciardi, Ugo Ramenghi, Ilaria Galliano, Massimiliano Bergallo

**Affiliations:** 1 Department of Pediatric Sciences and Public Health, University of Turin, Piazza Polonia 94, 10126 Turin, Italy; giu.pruccoli@gmail.com (G.P.); cristina.calvi@unito.it (C.C.); carla.alliaudi@unito.it (C.A.); marco.denina@gmail.com (M.D.); francesco.licciardi@gmail.com (F.L.); ugo.ramenghi@unito.it (U.R.); ilaria.galliano@unito.it (I.G.); 2 Infectious Diseases Unit, Department of Pediatrics, Regina Margherita Children’s Hospital, Piazza Polonia 94, 10126 Turin, Italy; silvia.garazzino@unito.it (S.G.); fede_by@hotmail.com (F.M.); carlo.scolfaro@unito.it (C.S.); marisazoppo@gmail.com (M.Z.); 3 Pediatric Laboratory, Department of Pediatric Sciences and Public Health, University of Turin, 10126 Turin, Italy; valentina.dapr@yahoo.it

**Keywords:** SARS-CoV-2, COVID-19, children, interferon, TRIM28, SETDB1, human endogenous retroviruses

## Abstract

Children with the new coronavirus disease 2019 (COVID-19) have milder symptoms and a better prognosis than adult patients. Several investigations assessed type I, II, and III interferon (IFN) signatures in SARS-CoV-2 infected adults, however no data are available for pediatric patients. TRIM28 and SETDB1 regulate the transcription of multiple genes involved in the immune response as well as of human endogenous retroviruses (HERVs). Exogenous viral infections can trigger the activation of HERVs, which in turn can induce inflammatory and immune reactions. Despite the potential cross-talks between SARS-CoV-2 infection and TRIM28, SETDB1, and HERVs, information on their expressions in COVID-19 patients is lacking. We assessed, through a PCR real time Taqman amplification assay, the transcription levels of six IFN-I stimulated genes, IFN-II and three of its sensitive genes, three IFN-lIIs, as well as of TRIM28, SETDB1, pol genes of HERV-H, -K, and -W families, and of env genes of Syncytin (SYN)1, SYN2, and multiple sclerosis-associated retrovirus (MRSV) in peripheral blood from COVID-19 children and in control uninfected subjects. Higher expression levels of IFN-I and IFN-II inducible genes were observed in 36 COVID-19 children with mild or moderate disease as compared to uninfected controls, whereas their concentrations decreased in 17 children with severe disease and in 11 with multisystem inflammatory syndrome (MIS-C). Similar findings were found for the expression of TRIM-28, SETDB1, and every HERV gene. Positive correlations emerged between the transcriptional levels of type I and II IFNs, TRIM28, SETDB1, and HERVs in COVID-19 patients. IFN-III expressions were comparable in each group of subjects. This preserved induction of IFN-λs could contribute to the better control of the infection in children as compared to adults, in whom IFN-III deficiency has been reported. The upregulation of IFN-I, IFN-II, TRIM28, SETDB1, and HERVs in children with mild symptoms, their declines in severe cases or with MIS-C, and the positive correlations of their transcription in SARS-CoV-2-infected children suggest that they may play important roles in conditioning the evolution of the infection.

## 1. Introduction

Severe acute respiratory syndrome coronavirus 2 (SARS-CoV-2), the etiologic agent of coronavirus disease 2019 (COVID-19), is a recently emerged coronavirus that has infected millions of people worldwide. COVID-19 is characterized by protean clinical pictures and different patterns of disease progression, including fatal outcomes. Children develop a milder illness and have a better prognosis than their adult counterparts; however the reasons for these differences are poorly understood and reports on the underlying biological mechanisms leading to severe clinical manifestations in childhood are lacking [1].

Viral recognition induces the production of interferons (IFNs), which trigger the transcription of IFN-stimulated genes (ISGs) that are ultimately responsible for various antiviral and immunomodulatory functions. There are three types of IFNs. Type I IFNs (IFN-Is: IFN-α, -β, -ε, -κ, -ω in humans) bind to the ubiquitously expressed receptor (IFNAR) and through a JAK-STAT pathway activate the transcription of thousands of genes [2]. Type I ISG products have immunomodulatory properties and are implicated in the development of the cytokine storm that can lead to fatal outcomes in COVID-19 patients [2,3,4]. The IFN-γ is the sole type II IFN (IFN-II), released by NK cells and mostly by Th1 cells, which also elicits the activation of thousands of genes [5]. CXCL9, CXCL10, and IDO1 are prevalently IFN-γ-induced chemokines. Their expressions correlate with the tissue infiltration of inflammatory cells, in particular of T cells [6]. A third type of interferons (IFN-III), also referred to as lambdas (IFNλs), in humans comprise four members: IFNλ1/IL-29, IFNλ2/IL-28A, IFNλ3/IL-28B, and IFNλ4. They bind to a unique heterodimeric receptor complex, which is restricted to epithelial cells and a subset of immune cells [7]. IFNλs play a major role in the antiviral protection of mucosa barriers [8] and exhibit lower pro-inflammatory properties than type I IFNs [9,10].

In cell cultures, all IFN types inhibit SARS-CoV-2 replication in a dose-dependent manner [11,12]. In vivo studies have also shown the importance of IFNs. Subjects harboring pathogenetic variants of genes involved in type I and III IFN immunity [13] or with high titers of neutralizing autoantibodies against IFN-Is [14] are more likely to have critical COVID-19 disease. Patients with mild to moderate symptoms mount a strong, early IFN response, whereas a weak or delayed IFN response is associated with extensive viral spread, a hyperinflammatory state, and the development of severe illness [15,16]. Due to such potent IFN-driven antiviral activities, many viruses have developed mechanisms to escape their effects [17,18]. For instance, some coronavirus proteins antagonize IFN-I functions [3,19,20]. Notably, no information is available on expression of IFNs and ISGs in COVID-19 children.

The induction of IFNs may be regulated by environmental factors via epigenetic mechanisms, such as DNA methylation and heterochromatin-silencing by histone modifications. Krüppel-associated box domain zinc finger proteins (KRAB-ZFPs) are the largest family of transcriptional regulators in the human genome [21]. Tripartite motif containing 28 (TRIM28), also known as KAP1 or TIF1-β, is considered a universal nuclear corepressor of KRAB-ZFPs [22]. SET domain bifurcated histone lysine methyltransferase 1 (SETDB1), also known as ESET, is a histone H3K9 methyltransferase that contributes with TRIM28 and KRAB-ZFPs to heterochromatin formation [23]. Recent studies have drawn attention to the involvement of TRIM28 and SETDB1 in many aspects of cell homeostasis and in epigenetic control of the immune response [24,25,26], including antiviral response and IFN production [27,28].

TRIM28 and SETDB1 were uncovered mainly through researches on host factors that repress transcription of endogenous retroviruses [29]. These constitute about 8% of our genome. They originate from ancestral infections that led to their integration into the DNA of primates over 25 million years ago [30]. During evolution, the accumulation of mutations blocked the production of infectious virions, but some viral sequences are transcribed and a few encode proteins, such as Syncytin-1 (SYN1) [31] and Syncytin-2 (SYN2) [32], that have been co-opted for crucial physiologic functions, such as placenta morphogenesis and feto-maternal immune tolerance [33]. Human endogenous retroviruses (HERVs) maintain a typical retroviral structure with three principal genes: group-associated antigens (gag), polymerase (pol), and envelope (env), flanked between two regulatory long terminal repeats (LTRs). HERVs are extensively distributed throughout the genome and are able to regulate the transcription of close cellular genes [34]. HERV RNAs through retro-transposition can generate novel insertions into DNA and, being sensed as non-self by pattern recognition receptors (PRRs), they can elicit inflammatory and immune responses [34,35,36,37]. Some viral proteins, such as the HERV-W-env protein of MSRV (multiple sclerosis-associated retrovirus), can trigger autoimmunity [38,39], while others, such as the syncytins, exhibit intrinsic immunomodulatory activity [40,41,42]. Aberrant HERV expressions are associated with inflammatory and immune-mediated diseases, supporting their potential etiopathogenetic role in these pathologies [39,43,44,45]. External viral infections can induce HERV transcription [46,47,48,49,50,51] and IFNs and inflammatory cytokines lead to the independent and synergistic activation of retroviral sequences [52]. In cultured cells, the expression of retroviral elements was significantly up-regulated upon infection with SARS-CoV-2 [53,54]. Despite the potential mutual interplays between TRIM28/SETDB1, HERVs, and SARS-CoV-2, no investigations have explored their activation in patients affected by COVID-19, but a recent report that has highlighted the higher expression of MSRV-env in adults [55].

In the present study we assessed the transcriptional levels of (1) type I, II, and III IFNs and/or of their ISGs, (2) TRIM28 and SETDB1, and (3) HERV sequences of env genes of SYN1, SYN2, MSRV, and of pol genes of HERV-H, -K, -W, the three retroviral families most widely studied, in children with mild/moderate or severe clinical manifestations of COVID-19 and in uninfected control children.

## 2. Results

### 2.1. Study Populations

The study population was divided in four groups. A total of 64 patients with SARS-CoV-2 infection (37 males, median age 7.8 years, IQR 2.3; 12.5 years) were enrolled in the study. Of these, Group A included 36 children with mild/moderate symptoms, Group B 17 patients with severe clinical manifestations, and Group C 11 patients with severe multisystem inflammatory syndrome in children (MIS-C). Group D included: 60 uninfected control children (37 males, median age 4.91 years, IQR 2.2; 9.1 years) for the detection of type I, II, and III IFN signatures, TRIM28 and SETDB1; 49 uninfected children for the detection of env genes of SYN1, SYN2, and MSRV (34 males, median age 4.5 years, IQR 1.7; 13.0 years); and 108 children (57 males, median age 5.2 years, IQR 3.5; 12.6 years) for the detection of pol genes of HERV-H, HERV-K, and HERV-W who were investigated as a control group in our previous studies [45,51,56].

### 2.2. Characteristics of Infected Children

As detailed in Table 1, the time interval between symptom onset and blood sample collection was longer in Group C children than in Group A and Group B children, reflecting in part the latency period before overt MIS-C from initial infection. However, in most Group C children, primary SARS-CoV-2 infection went unnoticed and first symptoms were related to MIS-C.

Among the 27 children with comorbidities, five had malignancies, five hemoglobin diseases, two cystic fibrosis, and one diabetes. 

Supplemental oxygen was administered to seven Group B and six Group C patients. Two Group B patients and two Group C patients were admitted to the ICU. Biochemical signs of hepatitis were present in two children. Thrombotic complications and multiorgan failure were observed in one child with concomitant pneumococcal sepsis. 

All Group C patients developed cardiac dysfunctions; one had long-lasting coronary dilatations.

Group B and C patients had higher markers of systemic inflammation, such as C-reactive protein (CRP), erythrocyte sedimentation rate (ESR), procalcitonin (PCT), and ferritin. 

At time of testing, 27 patients (43%) had received varying combinations of antimicrobials; prophylactic or therapeutic anticoagulation had been administered to five Group B and seven Group C patients; and IV IgG to one group C patient. Eleven (65%) Group B and eleven (100%) Group C patients were under steroid treatment at the time of sampling.

At follow-up, no Group A patient developed severe symptoms or MIS-C and no patient died.

### 2.3. Type I IFN Signature 

**Figure 1:**
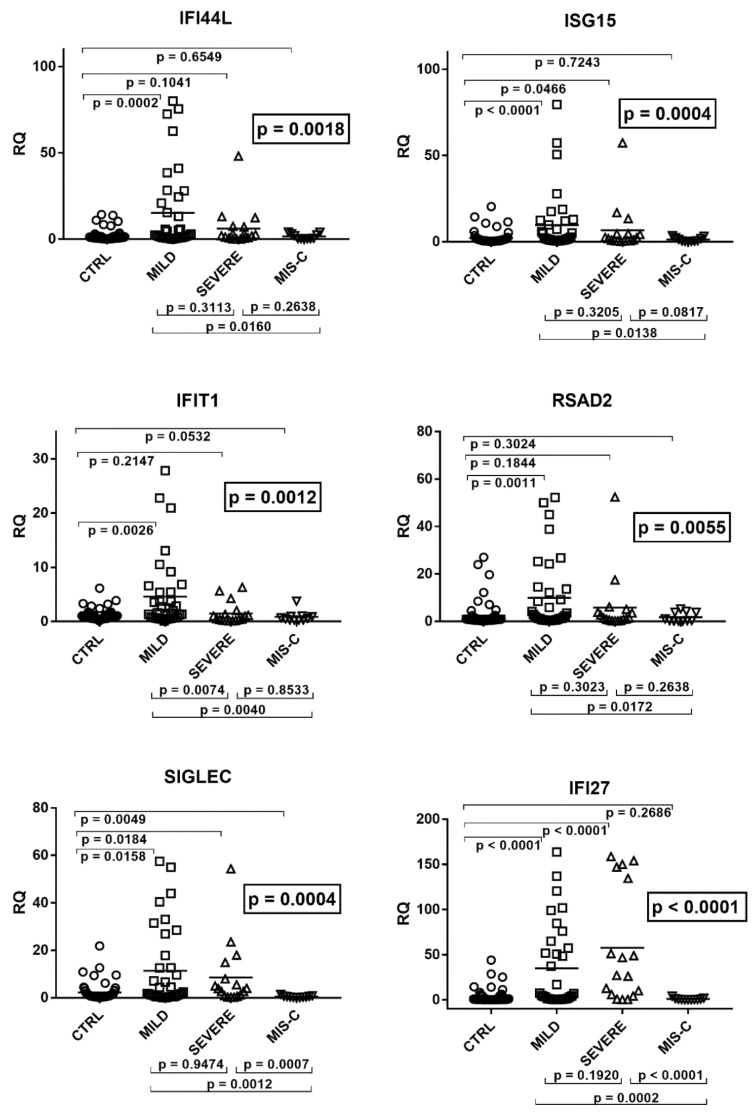
Expression of type I interferon stimulated genes (ISGs) in whole blood from 64 children with SARS-CoV-2 infection and 60 uninfected children. CTRL: uninfected control children. MILD: infected children with mild/moderate symptoms. Severe: infected children with severe disease. MIS-C: infected children with multisystem inflammatory syndrome. RQ: relative quantification. Circles, squares, and triangles show the mean of three individual measurements; horizontal lines the median values. Median values and interquartile range (IQR) of six ISGs: IFI44L: CTRL: median 0.91, IQR 0.47, 1.65; MILD: median 2.27, IQR 0.88, 21.76; SEVERE: median 1.95, IQR 0.77, 7.06; MIS-C: median 0.52, IQR 0.24, 2.80. ISG15: CTRL: median 0.74, IQR 0.48, 1.73; MILD: median 1.85, IQR 0.97, 10.37; SEVERE: median 1.85, IQR 0.66, 4.63; MIS-C: median 0.64, IQR 0.36, 2.11. IFIT1: CTRL: median 0.86, IQR 0.56, 1.36; MILD: median 1.57, IQR 0.69, 5.40; SEVERE: median 0.50, IQR 0.26, 1.31; MIS-C: median 0.57, IQR 0.29, 0.92. RSAD2: CTRL: median 0.77, IQR 0.51, 1.26; MILD: median 1.94, IQR 0.73, 12.52; SEVERE: median 1.44, IQR 0.65, 3.82; MIS-C: median 0.34, IQR 0.23, 3.83. SIGLEC: CTRL: median 0.78, IQR 0.46, 1.87; MILD: median 1.74, IQR 0.48, 13.91; SEVERE: median 3.90, IQR 0.87, 7.94; MIS-C: median 0.33, IQR 0.20, 0.62. IFI27: CTRL: median 0.56, IQR 0.27, 1.68; MILD: median 4.74, IQR 1.04, 57.36; SEVERE: median 26.98, IQR 5.78, 134,42; MIS-C: median 0.32, IQR 0.17, 0.96. Statistical analysis: one-way ANOVA was used to compare the transcriptional levels of each target between the four groups of children. The Mann–Whitney test was used to compare the transcriptional levels of each target between each group of children with each other.

Downstream signaling of IFN-Is differed significantly through ANOVA analysis between the four groups of children (Figure 1).

As illustrated in the figure, the medians of the six ISGs were significantly higher in infected children with mild symptoms than in the control group. A trend to higher values for most ISGs was observed also in children with severe disturbances vs. the control group, with significant differences for IFI27, ISG15, and SIGLEC. In contrast, patients with MIS-C exhibited a trend to lower values vs. the control group, with statistical significance for SIGLEC. Comparing the three groups of COVID-19 patients with each other, there was a trend to lower values for most ISGs in subjects with severe disturbances vs. subjects with mild symptoms, with a significant difference for IFIT1. This downregulation reached statistical significance for all ISGs by comparing MIS-C patients with the group with mild symptoms. The impaired expression of most ISGs in MIS-C patients emerged also when these were compared to those with severe disease, with statistical differences for SIGLEC and IFI27 and a borderline *p* value for ISG15 (Figure 1).

### 2.4. Type II IFN Signature

**Figure 2:**
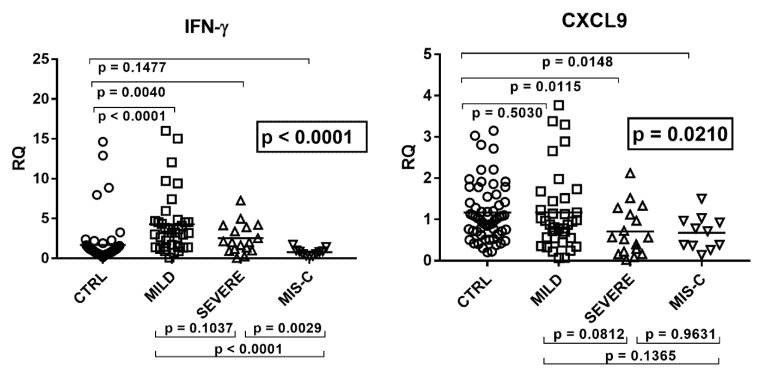

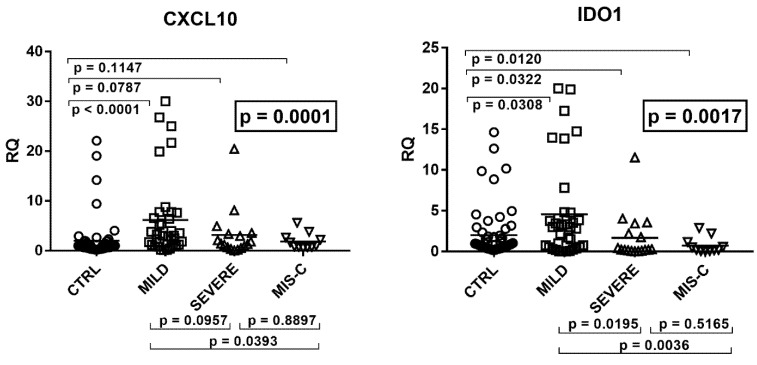
Expression of type II interferon and of CXCL9, CXCL10, and IDO1 in whole blood from 64 children with SARS-CoV-2 infection and 60 uninfected children. CTRL: uninfected control children. MILD: infected children with mild/moderate symptoms. Severe: infected children with severe disease. MIS-C: infected children with multisystem inflammatory syndrome. RQ: relative quantification. Circles, squares, and triangles show the mean of three individual measurements, horizontal lines the median values. Median values–interquartile range (IQR) of IFN-γ and its sensitive genes: IFN-γ: CTRL: median 0.98, IQR 0.56, 1.37; MILD: median 3.23, IQR 1.45, 4.59; SEVERE: median 2.02, IQR 1.17, 3.92; MIS-C: median 0.80, IQR 0.44, 0.93. CXCL9: CTRL: median 1.03, IQR 0.71, 1.58; MILD: median 0.91, IQR 0.55, 1.46; SEVERE: median 0.56, IQR 0.22, 1.12; MIS-C: median 0.71, IQR 0.37, 0.95. CXCL10: CTRL: median 0.78, IQR 0.51, 1.32; MILD: median 3.00, IQR 1.21, 6.82; SEVERE: median 1.41, IQR 0.67, 3.35; MIS-C: median 0.94, IQR 0.75, 2.40. IDO1: CTRL: median 0.82, IQR 0.43, 1.80; MILD: median 3.00, IQR 0.56, 4.23; SEVERE: median 0.24, IQR 0.15, 2.20; MIS-C: median 0.21, IQR 0.10, 0.86. Statistical analysis: one-way ANOVA was used to compare the transcriptional levels of each target between the four groups of children. The Mann–Whitney test was used to compare the transcriptional levels of each target between each group of children with each other.

As detailed in Figure 2, the transcriptional levels of IFN-γ and its sensitive genes differed significantly by ANOVA analysis among the four groups of children.

In particular, children with mild symptoms had significantly higher mRNA values of IFN-γ, CXCL10, and IDO1 as compared to the control group. Children with severe disturbances had higher values vs. the control group of IFN-γ and CXCL10, while CXCL9 and IDO1 expressions were decreased. In MIS-C patients mRNA levels of IFN-γ and CXCL10 did not differ significantly from healthy subjects, while CXCL9 and IDO1 values were significantly reduced. The comparison between COVID-19 patients showed that those with severe symptoms had borderline or significantly (for IDO1) lower values vs. those with mild symptoms. Children with MIS-C had significantly lower concentrations of IFN-γ transcripts as compared to both groups of COVID-19 children. They also exhibited lower levels of CXCL10 and IDO1 when compared to the group with mild symptoms (Figure 2). 

### 2.5. Type III IFNs

**Figure 3:**
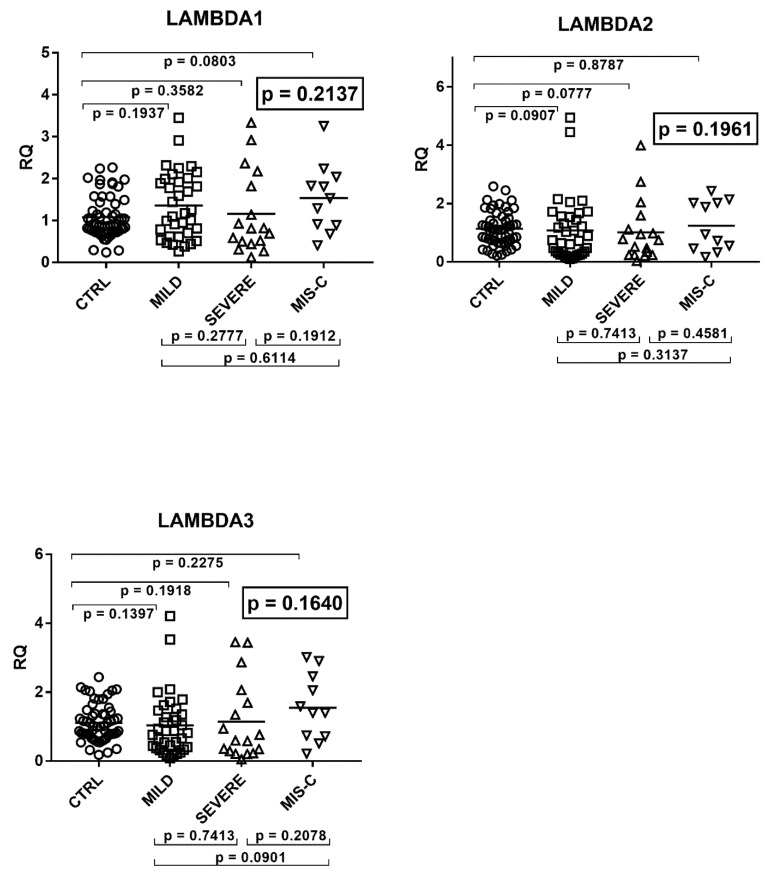
Expression of type III interferons in whole blood from 64 children with SARS-CoV-2 infection and 60 uninfected children. CTRL: uninfected control children. MILD: infected children with mild/moderate symptoms. Severe: infected children with severe disease. MIS-C: infected children with multisystem inflammatory syndrome. RQ: relative quantification. Circles, squares, and triangles show the mean of three individual measurements, horizontal lines the median values. Median values–interquartile range (IQR) of IFN-IIIs: LAMBDA1: CTRL: median 0.90, IQR 0.75, 1.40; MILD: median 1.17, IQR 0.66, 1.96; SEVERE: median 0.81, IQR 0.50, 1.82; MIS-C: median 1.53, IQR 0.90, 1.93. LAMBDA2: CTRL: median 1.11, IQR 0.74, 1.46; MILD: median 0.73, IQR 0.28, 1.54; SEVERE: median 0.75, IQR 0.24, 1.11; MIS-C: median 0.94, IQR 0.50, 2.02. LAMBDA3: CTRL: median 1.00, IQR 0.77, 1.38; MILD: median 0.84, IQR 0.40, 1.38; SEVERE: median 0.60, IQR 0.29, 1.69; MIS-C: median 1.40, IQR 0.73, 2.25. Statistical analysis: one-way ANOVA was used to compare the transcriptional levels of each target between the four groups of children. The Mann–Whitney test was used to compare the transcriptional levels of each target between each group of children with each other.

The transcriptional levels of IFN-λ1, IFN-λ2, and IFN-λ3 did not differ significantly by ANOVA analysis in the four groups of children (Figure 3). In particular, there were no significant differences between each group of infected children and the control group, as well as between each group of COVID-19 patients.

### 2.6. Expressions of TRIM28 and SETDB1

**Figure 4:**
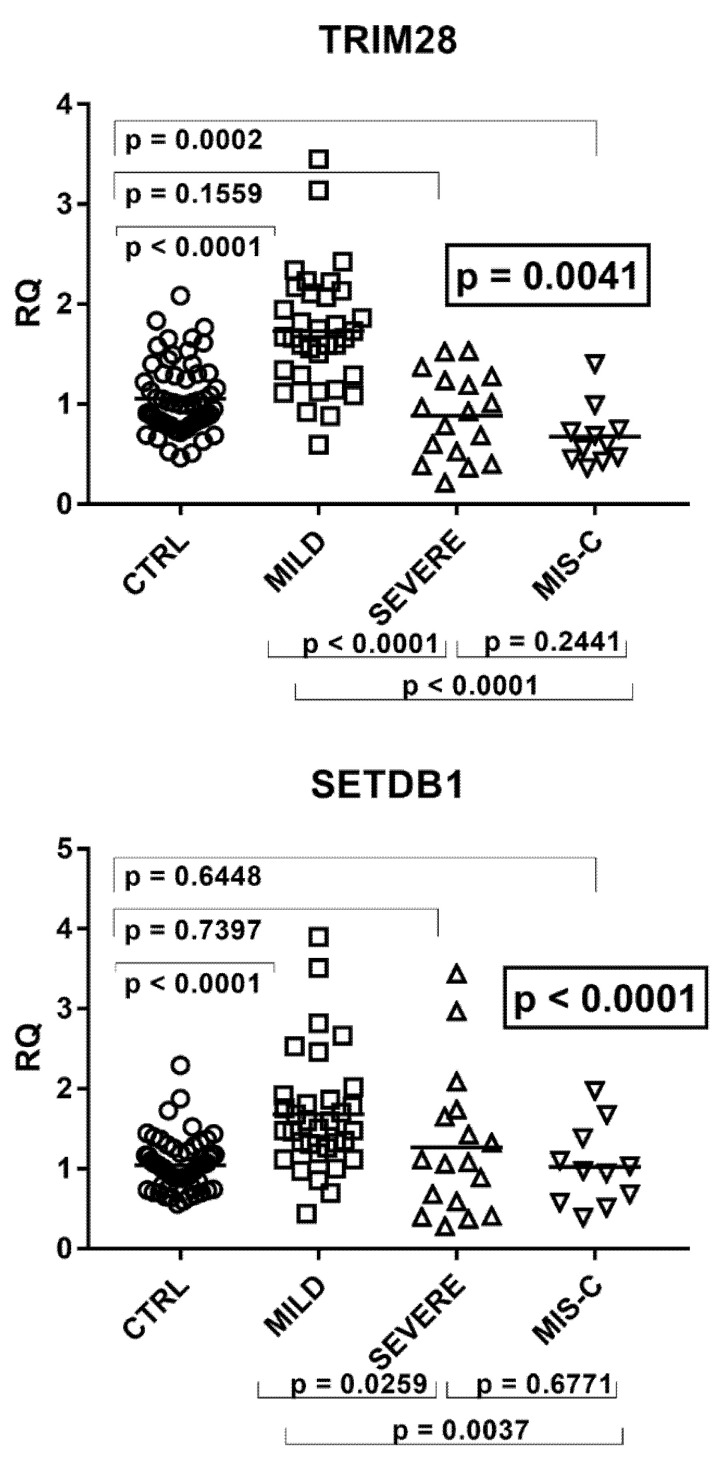
Expression of TRIM28 and SETDB1 in whole blood from 64 children with SARS-CoV-2 infection and 60 uninfected children. CTRL: uninfected control children. MILD: infected children with mild/moderate symptoms. Severe: infected children with severe disease. MIS-C: infected children with multisystem inflammatory syndrome. RQ: relative quantification. Circles, squares, and triangles show the median of three individual measurements, horizontal lines the median values. Median values–interquartile range (IQR): TRIM28: CTRL: median 1.00, IQR 0.80, 1.30; MILD: median 1.66, IQR1.29, 2.07; SEVERE: median 0.93, IQR 0.53, 1.24; MIS-C: median 0.60, IQR 0.46, 0.74. SETDB1: CTRL: median 1.00, IQR 0.80, 1.19; MILD: median 1.50, IQR 1.27, 1.87; SEVERE: median 1.08, IQR 0.59, 1.65; MIS-C: median 0.97, IQR 0.63, 1.24. Statistical analysis: one-way ANOVA was used to compare the transcriptional levels of each target between the four groups of children. The Mann–Whitney test was used to compare the transcriptional levels of each target between each group of children with each other.

The transcriptional levels of TRIM28 and SETDB1 differed significantly among the four groups of children (Figure 4).

As detailed in the figure, transcriptional levels of both TRIM28 and SETDB1 were higher in children with mild symptoms than in the control group, while their levels decreased in patients with severe clinical pictures or MIS-C as compared to patients with mild symptoms.

### 2.7. Correlations between Expressions of IFNs and ISGs and TRIM28 or SETDB1

**Figure 5:**
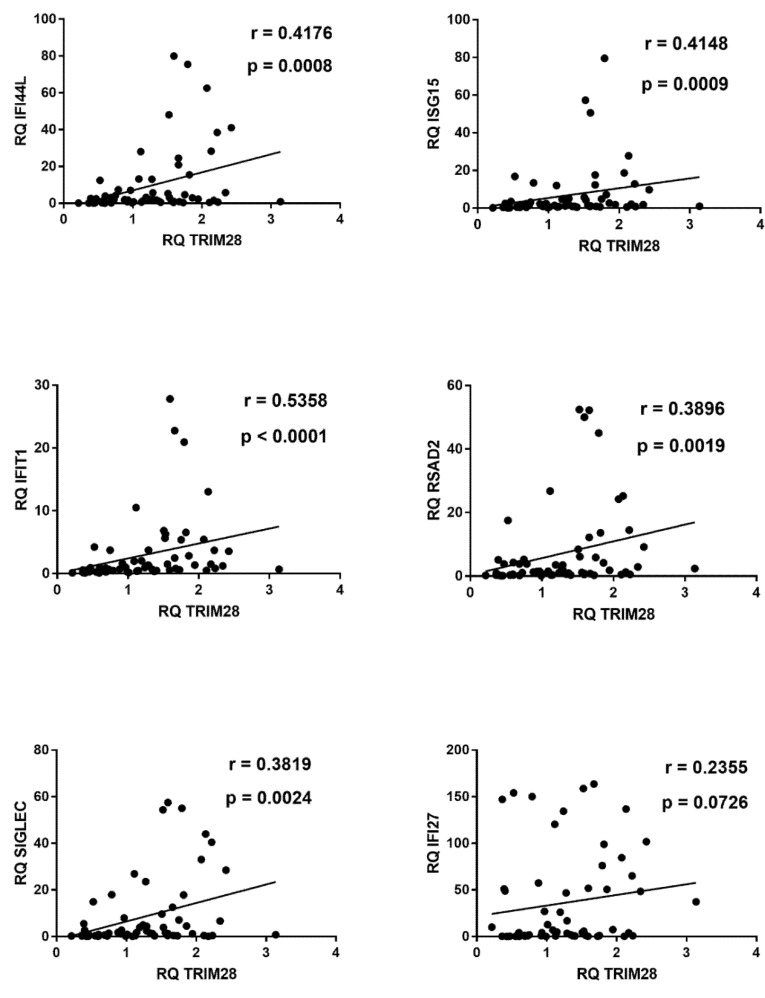
Correlations between transcription levels of type I ISGs and TRIM28 in whole blood from 64 COVID-19 children. RQ: relative quantification. Circles show the mean of three individual measurements. Line: linear regression line. Statistical analysis: Spearman correlation test.

In COVID-19 children significant positive correlations were found between expressions of type I ISGs and TRIM28 (Figure 5).

**Figure 6:**
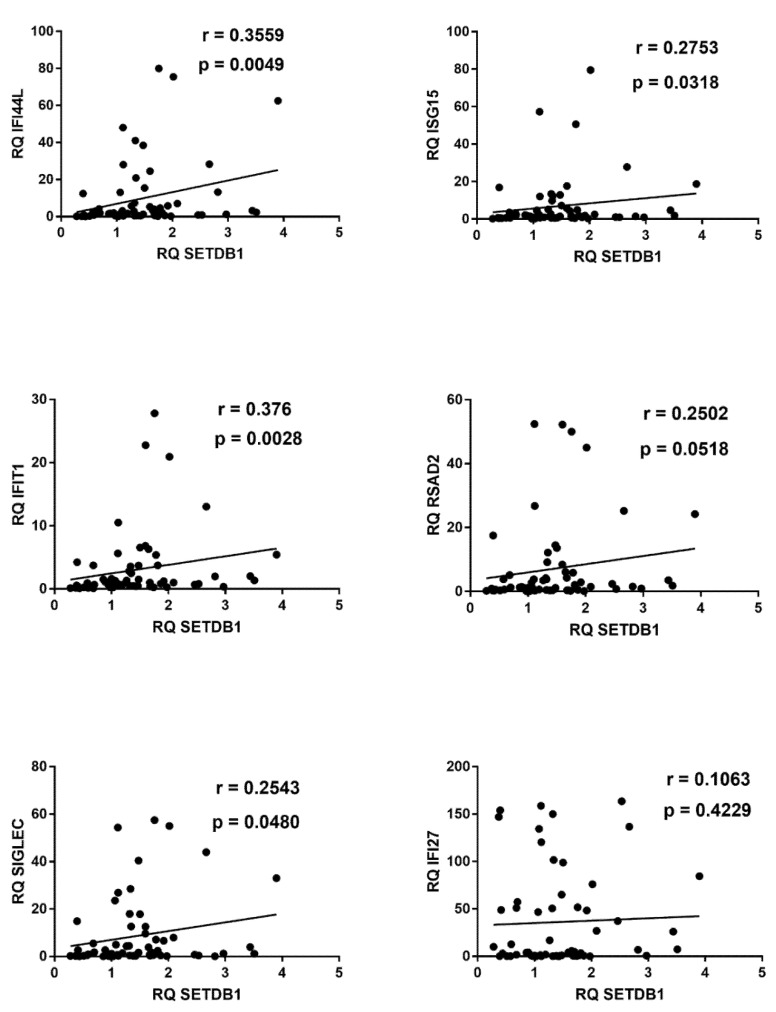
Correlations between transcription levels of type I IFNs and SETDB1 in whole blood from 64 COVID-19 children. RQ: relative quantification. Circles show the mean of three individual measurements. Line: linear regression line. Statistical analysis: Spearman correlation test.

Similar positive correlations were found between IFN-I ISGs and SETDB1, with the exception of IFI27 (Figure 6).

**Figure 7:**
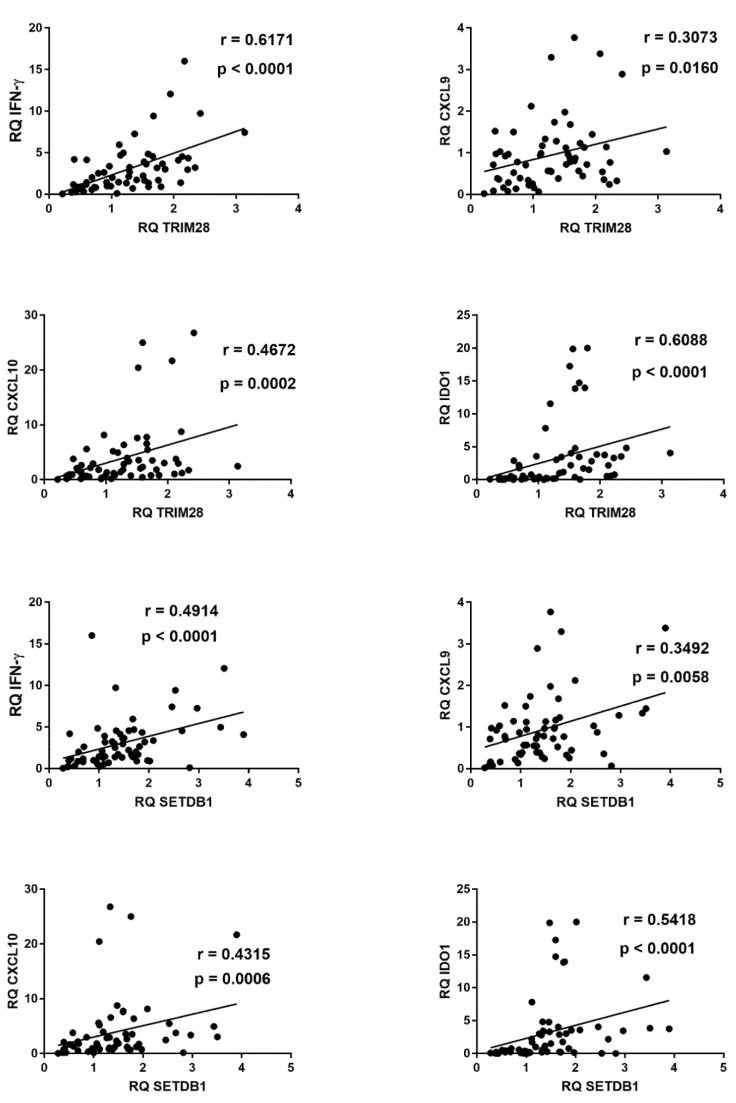
Correlations between transcription levels of type II IFN and its sensitive genes and TRIM28 or SETDB1 in whole blood from 64 COVID-19 children. RQ: relative quantification. Circles show the mean of three individual measurements. Line: linear regression line. Statistical analysis: Spearman correlation test.

Significant positive correlations emerged also between transcription levels of IFN-γ, CXCL9, CXCL10, IDO1, and of TRIM28 or SETDB1 (Figure 7).

In contrast, in control children no significant correlations were found between transcription levels of type I ISGs, IFN-γ, CXCL9, CXCL10, IDO1, and IFN-λs vs. mRNA concentrations of TRIM28 or SETDB1 (data not shown).

### 2.8. Expressions of HERV-H-pol, HERV-K-pol, HERV-W-pol, and of SYN1-env, SYN2-env, and MSRV-env

**Figure 8:**
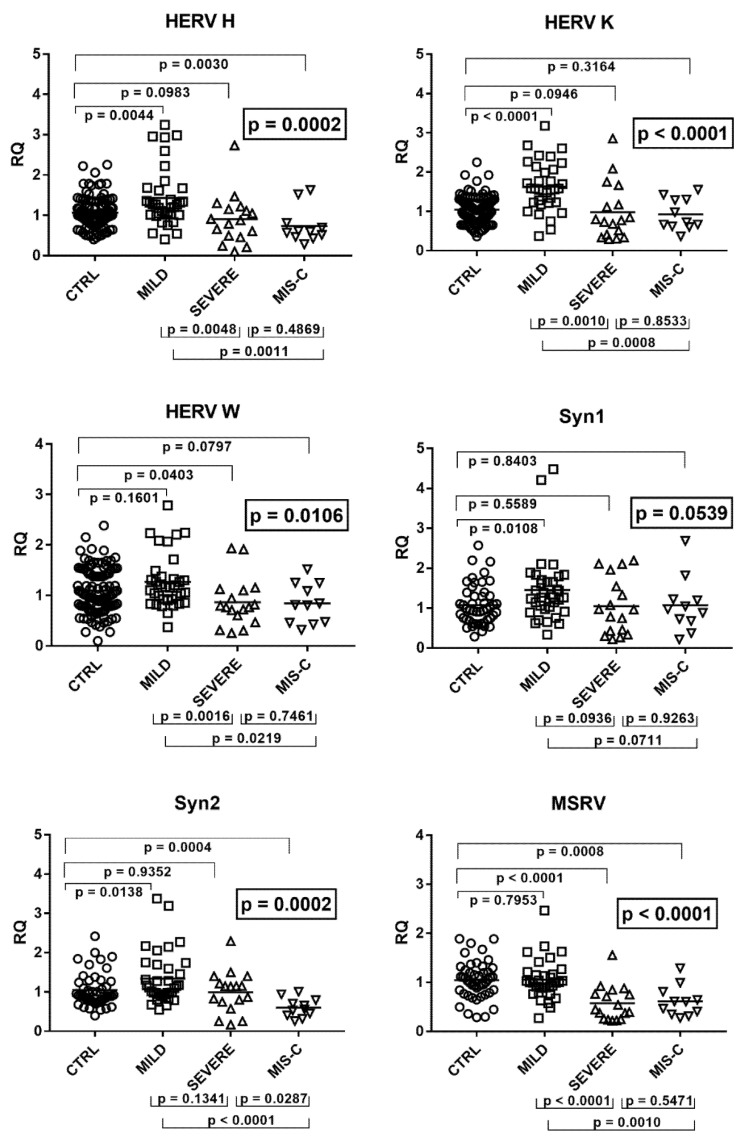
Transcription levels of pol genes of HERV-H, HERV-K, and HERV-W, and of env genes of SYN1, SYN2, and MSRV in whole blood from 64 children with SARS-CoV-2 infection and 108 uninfected children for HERV-pols and 49 uninfected children for SYN1-env, SYN2-env, and MSRV-env. CTRL: uninfected control children. MILD: infected children with mild/moderate symptoms. Severe: infected children with severe disease. MIS-C: infected children with multisystem inflammatory syndrome. RQ: relative quantification. Circles, squares, and triangles show the median of three individual measurements, horizontal lines the median values. Median values–interquartile range (IQR): HERV-H-pol: CTRL: median 1.01, IQR 0.80, 1.32; MILD: median 1.27, IQR 1.02, 1.64; SEVERE: median 0.88, IQR 0.49, 1.15; MIS-C: median 0.59, IQR 0.49, 0.75. HERV-K-pol: CTRL: median 1.05, IQR 0.80, 1.30; MILD: median 1.57, IQR 1.23, 2.05; SEVERE: median 0.77, IQR 0.41, 1.17; MIS-C: median 0.72, IQR 0.64, 1.30. HERV-W-pol: CTRL: median 1.03, IQR 0.79, 1.44; MILD: median 1.15, IQR 0.91, 1.35; SEVERE: median 0.77, IQR 0.61, 1.10; MIS-C: median 0.81, IQR 0.47, 1.17. Syncytin 1-env: CTRL: median 0.98, IQR 0.70, 1.39; MILD: median 1.28, IQR 1.00, 1.68; SEVERE: median 0.96, IQR 0.43, 1.54; MIS-C: median 0.97, IQR 0.70, 1.21. Syncytin 2-env: CTRL: median 0.91, IQR 0.77, 1.27; MILD: median 1.12, IQR 0.94, 1.65; SEVERE: median 1.14, IQR 0.75, 1.21; MIS-C: median 0.56, IQR 0.43, 0.75. MSRV-env: CTRL: median 1.05, IQR 0.80, 1.24; MILD: median 1.00, IQR 0.89, 1.16; SEVERE: median 0.45, IQR 0.26, 0.76; MIS-C: median 0.60, IQR 0.38, 0.73. Statistical analysis: one-way ANOVA was used to compare the transcriptional levels of each target between the four groups of children. The Mann–Whitney test was used to compare the transcriptional levels of each target between each group of children with each other.

The mRNA levels of pol genes of HERV-H, -K, and -W as well as of env genes of SYN1, SYN2, and MSRV differed significantly among the four groups of children (Figure 8).

As illustrated in the figure, children with mild symptoms had mostly higher values vs. those of the control group, reaching the statistical significance for HERV-H-pol, HERV-K-pol, SYN1-env, and SYN2-env. In contrast, children with severe symptoms had significantly lower transcripts for HERV-W-pol and MSRV-env with borderline values for HERV-H-pol and HERV-K-pol as compared to the control group. MIS-C children had a significantly impaired transcriptions of HERV-H-pol, SYN2-env, and MSRV-env, with a borderline *p* value for HERV-W pol, vs. the control group. The comparison between COVID-19 patients showed that children with mild symptoms had higher mRNA concentrations of HERV genes than those with severe complications (significantly for all, but SYN1 and SYN2) or MIS-C (significantly for all, but borderline values for SYN1). No significant difference was found between severe cases vs. MIS-C patients but for SYN2, which was lower in MIS-C (Figure 8).

### 2.9. Correlations between Expressions of HERVs and TRIM28 or SETDB1 

**Figure 9:**
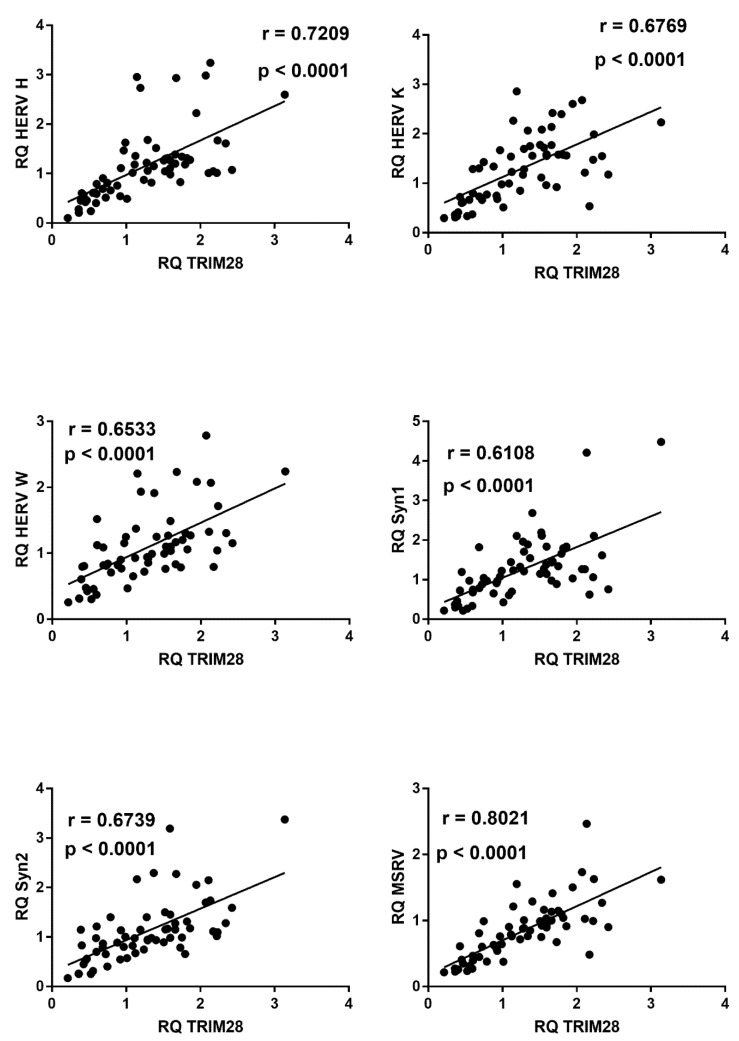
Correlations between transcription levels of TRIM28 and HERV sequences in whole blood from 64 COVID-19 children. RQ: relative quantification. Circles show the mean of three individual measurements. Line: linear regression line. Statistical analysis: Spearman correlation test.

**Figure 10:**
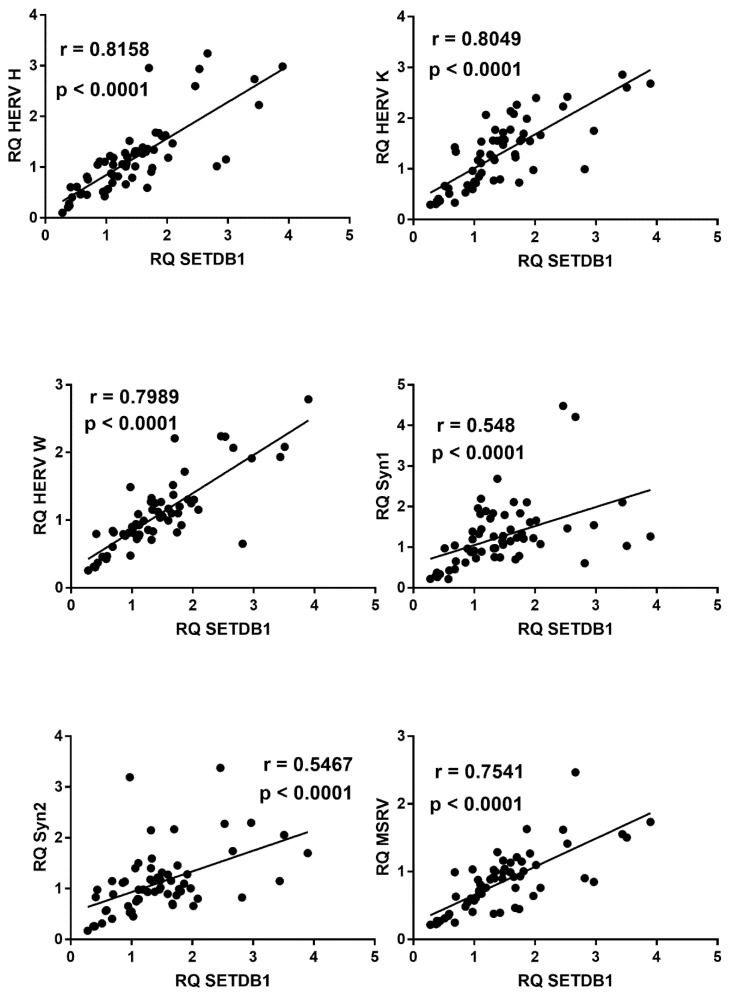
Correlations between transcription levels of SETDB1 and HERV sequences in whole blood from 64 COVID-19 children. RQ: relative quantification. Circles show the mean of three individual measurements. Line: linear regression line. Statistical analysis: Spearman correlation test.

In COVID-19 children, mRNA levels of all HERV sequences strongly correlated with the levels of TRIM28 (Figure 9) or SETDB1 (Figure 10), whereas no significant correlations were found between these variables in healthy children (data not shown).

### 2.10. Correlations between Expressions of IFNs or ISGs and HERV Sequences

In COVID-19 children, significant direct correlations were found in most analyses between mRNA levels of single IFN-I ISGs or of IFN-II and its sensitive genes vs. those of HERV-H-pol, HERV-K-pol, HERV-W-pol, SYN1-env, SYN2-env, and MSRV-env (a few examples are illustrated in Figures S1 and S2 added as supplement). In contrast, no significant correlations were observed in control children.

## 3. Discussion

An extensive body of literature underlines the importance of IFNs for the optimal control of SARS-CoV-2 infection, though several studies point to their potential involvement in the immunopathology of severe COVID-19. We performed a targeted analysis on IFNs and ISGs in a cohort of COVID-19 children with various clinical phenotypes and disease severity. There is a general agreement on the crucial role of type I and type III IFNs during early phases of viral infections. Our results highlight that IFN-I signature, based on the expression of six ISGs, was significantly higher in children with mild symptoms than in control subjects. Significant declines in IFN-I ISG score emerged with the development of severe clinical manifestations or MSIC-C, with marginal variations between these two groups of patients. Our findings are consistent with most reports in COVID-19 adults documenting high IFN-I expressions in mildly symptomatic subjects, while their deficiency was a hallmark of severe disease, linked to a persistent viral load and a hyperstimulation of the inflammatory response [16,57,58,59,60,61,62,63], even if contrasting results have been reported [64,65]. 

SARS-CoV-2 infection did not cause significant variations in IFN-IIIs expression in any group of children, whereas impaired IFN-λ activation was observed in adult patients with severe or fatal complications [63,66]. IFN-IIIs act together with IFN-I to fine-tune the IFN-driven innate immune response against viral infections and to guarantee a valid protection with minimal collateral damages. IFN-λs are actually able to prevent viral replication, but also to block the development of cytokine storm and IFN-I-driven immunopathology [9,67,68]. Whether the preserved IFN-III production contributes to the better evolution of SARS-CoV-2 infection in the pediatric age is an intriguing hypothesis that remains to be verified in large representative populations. 

Both type I and III IFNs have been proposed and are under investigation as therapeutic interventions in COVID-19 patients [69] (NCT04385095, NCT04331899, NCT04343976). Should these trials demonstrate positive results in adults, based on our findings one would expect similar positive effects also in pediatric age using type I IFNs, but irrelevant advantages with administration of type III IFNs, given their normal expressions in infected children irrespective of disease severity.

IFN-γ is a cytokine released mainly during the adaptive immune response by T helper cells. It was described to be involved, along with other pro-inflammatory cytokines, in tissue damage and mortality in COVID-19 patients [70]. Increased amounts of IFN-γ and CXCL10 have been associated with the disease severity [71], although with controversial evidence [65,72]. Based on the putative detrimental effects of IFN-γ, trials using anti-IFN-γ emapalumab and other anti-inflammatory drugs have been proposed (NCT04324021). Our results document higher expressions of IFN-II and its sensitive genes in mildly symptomatic patients, while they showed a tendency to decrease in children with severe clinical pictures, particularly in MIS-C. The different variations in the same disease conditions of single genes thought to be induced mainly by IFN-γ may be due to the action of other factors involved in their transcription. An enhanced expression of IFN-γ in COVID-19 children may thus be an index of valid immune response, while its impaired production in severe cases does not support the use of anti-IFN-γ therapy in critical children. 

Coronaviruses induce profound epigenetic alterations in the epithelial and immune cells of the host upon infection [73]. Growing data have shed light on the pivotal roles of TRIM28 and SETDB1 in epigenetic regulation on a large array of immune functions. The TRIM protein family encompasses more than 70 members in humans. Many members are involved in the control of viral infections, either as direct antiviral restriction factors or through regulating immune signaling [74,75,76]. TRIM28 is a small ubiquitin-related modifier (SUMO) that through conjugation to lysine residues of target protein substrates causes their phosphorylation, ubiquitination, and proteasome-driven degradation. TRIM28 recruits SETDB1 for SUMOylation, a crucial transient post-translational event involved in essential cell functions such as transcriptional repression, RNA splicing, and protein degradation [77,78]. Ubiquitination changes in viral proteins and in host proteins were observed in SARS-CoV-2-infected cells [79]. Interestingly, the ubiquitination of the ACE2 receptor by E3 ligases leads to its degradation [80,81]. TRIM28 is an E3 ligase, and its higher expression in children with mild symptoms could thus contribute to the downregulation of the major SARS-CoV-2 entry receptor, in contrast to the impaired activation of TRIM28 in severe cases. TRIM28 and SETDB1 exert relevant regulatory activities in the induction of IFNs [27,28,82]. Their importance on adaptive immunity is also increasingly recognized. TRIM28 modulates the differentiation of T cells and their expansion into helper and regulatory phenotypes [25,83]. Enhanced TRIM28 expression, as emerged in mildly symptomatic children, represses inflammatory genes, while its defective activation, as in severe patients, gives rise to the expansion of DCs and enhanced T cell priming toward inflammatory effector cells [84]. TRIM-28-defective regulatory T cells fail to control autoimmune manifestations, whose increase in severe COVID-19 patients could thus be supported by the upstream downregulation of TRIM28 [85]. It is also strictly associated with the maintenance and renewal of stem cells [86] whose alterations may lead to the hematologic disorders frequently observed in severe COVID-19 patients [87]. SETDB1 has multifaced biological activities. It controls the Th1 gene network and ensures Th2 cell stability via its action on set of genes involved in immune response [88]. Intestinal symptoms are among the most frequent disturbances in MIS-C and were present in almost all of our MIS-C patients. SETDB1 is essential for intestinal epithelial homeostasis and the prevention of local inflammation [89], while its decreased expression, as in MIS-C, may promote bowel inflammation [90]. Briefly, our findings suggest that aberrant expressions of TRIM28 and SETDB1 in COVID-19 children may condition the evolution of the infection via their crucial regulatory functions on the immune system, from the innate response to the adaptive response. 

Unexpected results of our study were the significant positive correlations between the transcription levels of TRIM28 or SETDB1 and those of type I and type II IFNs. Given the regulatory functions of TRIM28/SETDB1 on IFN induction, it is tempting to speculate that they are the *primum movens* of such correlations. However, several lines of research demonstrate that they are universal corepressors that display a negative impact on the transcription of thousands of genes, including IFNs [28]. Therefore, the TRIM28/SETDB1 suppressive effects cannot explain the direct correlations with IFN signatures. On the other hand, positive influences of IFN-Is and IFN-II on TRIM expression have been described [91]. The variations in IFN induction during different disease phases might thus justify the parallel changes in TRIM28 and SETDB1 transcripts. Notably, no significant correlations were found when IFN levels were within the normal range as in control subjects. An alternative hypothesis of other regulatory pathways triggered by the virus and leading to simultaneous up- and down-regulation in transactivation of both systems cannot be excluded. 

Our study evidences, for the first time, that COVID-19 is associated with abnormal transcription levels of several retroviral sequences. In particular, HERV mRNA concentrations were mostly higher in children with mild symptoms than in healthy children, while the development of severe manifestations coincided with their down-regulation. The underlying biochemical mechanisms responsible for these alterations remain to be elucidated. Several exogenous viral infections can elicit HERV activation [46,47,48,49,50,51], with the transcription of a large number of HERV loci and the simultaneous upregulation of neighboring genes, with many of such neighboring genes being IFN-stimulated genes [92]. Influenza A virus induces a SUMO-mediated metabolic switch with modification status of TRIM28 resulting in the derepression of retroviral elements [50]. Overwhelming evidence indicates that the enhanced activation of TRIM28 and SETDB1 results in higher heterochromatin formation ultimately leading to HERV silencing. However, also in this case we surprisingly found highly significant positive correlations between TRIM28/SETDB1 and HERVs. IFNs are able to stimulate HERV transcription [52,93]. The fact that IFN-I and IFN-II mRNAs were directly related to HERV concentrations in COVID-19 children, while such correlations were not observed in uninfected children, supports the potential role of type I and type II IFNs in upregulating HERV trans-activations too.

The clinical impact of abnormal HERV expressions in COVID-19 children remains questionable. SARS-CoV-2 RNAs can be reverse-transcribed and integrated into the human genome [53]. HERV-pols are an important source of reverse transcriptase. Their hyperexpression could thus contribute to retro-transpose and integrate SARS-CoV-2 RNAs into the genome. This could account for the prolonged virus detection in oropharyngeal samples of infected subjects using highly sensitive PCR assays without representing true reinfection [94,95]. Patients with severe COVID-19 symptoms have a defective virus control, a massive inflammatory response, and are susceptible to autoimmune disorders. Endogenous retroviruses may contribute on the one side to the host defense and on the other side to the triggering of immune-mediated damage [44] with alterations of pivotal immune functions, such as the activation of inflammasome [36]. SYN1 inhibits antiviral responses and increases virus-induced inflammation [96]. It contributes to the production of chemokines, cytokines [42,97], and of the C-reactive protein via the TLR3/IL-6 pathway [98]. SYN2 participates in T cell-mediated immunosuppression [41]. Clinical observations have frequently identified neurological and autoimmune manifestations in COVID-19 patients [99]. MSRV-env can trigger brain inflammation and autoimmunity [39] and the association between HERV overexpression and autoimmune disorders is widely documented [34,35,36,37,45]. In this context it must however be underlined that HERV activation was higher in children with mild symptoms, while it mostly decreased in severe cases, when HERV concentrations were sometimes lower even in comparison to healthy children. Presumably, the potential negative impacts of HERV on cell homeostasis have to be kept under control to avoid adverse side effects. Furthermore, we evaluated the HERV transcriptional profiles, not their encoded proteins, and the enhanced expression of the MSRV-env protein was detected in the peripheral leucocytes of SARS-CoV-2-infected adults, including patients with severe respiratory disorders [55].

The biological markers taken in consideration in our study could be influenced not only by different phases of the disease, but also by its duration and most of all by the receipt of immunomodulatory drugs [64,65]. The time intervals of hospitalization and sampling from symptom onset were longer in MIS-C patients. Furthermore, the majority of Group B and all Group C patients were treated with corticosteroids that modulate the transcription of hundreds of genes involved in inflammatory and immune responses, including IFNs [100,101] and retroviral elements [102,103]. No children with mild or moderate symptoms progressed to severe clinical pictures, thus no longitudinal analysis to identify reliable prognostic markers over time could be performed. 

Our COVID-19 patients with mild symptoms had enhanced transcriptions of both host genes and retroviral sequences. The same genes were significantly downregulated in severe cases, sometimes reaching values below the physiological homeostasis levels. This could be due to an exhaustion of the virus-driven stimulatory mechanisms and/or the upregulation of specific inhibitory checkpoints, occurring through epigenetic changes in the landscape of the cell genome [104,105,106]. For instance, sterile alpha motif and HD-domain–containing protein 1 (SAMHD1) is able to inhibit IFN-I induction and block the activation of retroviruses [107]. IFNs are an example of highly active molecules whose excessive production can harm the host. Rapid and potent activation of counterregulatory pathways are consequently needed [78,108]. Whereas limited IFN-I and IFN-II exposures generate transcriptional memory and the maturation of somatic and hemopoietic stem cells [5], these are exhausted by repeated IFN stimulations [109,110]. Patients with COVID-19 pneumonia display T cell exhaustion and skewing towards TH17 inflammatory phenotype [111]. TRIM28/SETDB1-mediated SUMOylation is a highly dynamic process that can be removed rapidly by a family of specific deconjugating proteases [112,113]. Transcriptional and epigenetic studies have demonstrated that virus-induced exhaustion may be a long-lasting condition [104] and increasing evidence points to the prolonged persistence of complications that can affect every organ system after the acute phase of COVID-19 [114,115]. Whether an exhaustion of the immune response and/or excessive suppressive pathways have a role in worsening the disease course and in its long-term complications or represent a clinically irrelevant epiphenomenon requires further targeted studies. 

In conclusion, our study evidences enhanced transcription levels of IFN-I ISGs in COVID-19 children with mild symptoms and lower levels in severe cases. This further confirms the importance of type I IFNs for adequate control of the infection and supports their use also in pediatric age, if the ongoing trials in adults are to demonstrate their efficacy. In contrast to the deficiency in IFN-IIIs described in adults with severe disease, every group of our children exhibited similar levels of IFN-λ RNAs, raising the possibility that this difference contributes to the more favorable course of the infection in pediatric age. The impaired expression of IFN-II and its sensitive genes in severe patients argues again its crucial role in the cytokine storm characterizing the advanced disease phases and the therapeutic use of the anti-IFN-γ monoclonal antibody in children. The SARS-CoV-2-induced variations on TRIM28 and SETDB1 expressions may contribute to the altering of proper immune cell homeostasis and a valid host defense. The significant positive correlations between transcripts of IFN-I and IFN-II and of TRIM28 and SETDB1 suggest that the former may exert important regulatory functions on the transactivation of the latter in COVID-19 children. The variations in HERV expressions during different disease states add a new variable worth considering in the evolution of the infection. The simultaneous SARS-CoV-2-driven hyperexpression of cellular genes and provirus sequences in children with mild symptoms and their parallel declines with the development of severe clinical pictures suggest that immune exhaustion and/or suppressive counterregulatory mechanisms, aimed to control the damages of excessive or prolonged persistence of innate and adaptive immune responses, might have a role in conditioning the outcome of the infection. SARS-CoV-2 infection is characterized by several specificities in children as compared to adults. Present results highlight the trend of some variables in children with distinct phases of the disease, but their relevance may involve the overall COVID-19 topic, contributing to the identification of new prognostic markers and innovative therapeutic strategies.

## 4. Materials and Methods

### 4.1. Study Populations

Children who were admitted at the Regina Margherita Children’s Hospital, Turin, Italy, with laboratory-confirmed SARS-CoV-2 infection were enrolled in the study. The grade of disease severity was established according to previously reported criteria [116,117]. MIS-C was defined according to the CDC case definition (Centers for Disease Control and Prevention. Emergency Preparedness and Response: Multisystem Inflammatory Syndrome in Children (MIS-C) Associated with Coronavirus Disease 2019, COVID-19, 2020. Available online at: https://emergency.cdc.gov/han/2020/han00432.asp, accessed on 20 May 2020).

Uninfected healthy children tested at the same hospital before the beginning of the pandemics for routine laboratory examinations and whose results were all within normal limits were adopted as control group. Subjects with any confirmed or suspected disease, such as infections, cancer, autoimmune disorders, inflammatory diseases, neurological disturbances, or abnormal laboratory results were excluded from the study.

### 4.2. Total RNA Extraction

Total RNA was extracted from whole blood using the automated extractor Maxwell (Promega, Madison, WI, USA) following the RNA Blood Kit protocol without modification. This kit provides treatment with DNase during the RNA extraction process. The RNA concentration and purity were assessed by traditional UV spectroscopy with absorbance at 260 and 280 nm. The nucleic acid concentration was calculated using the Beer–Lambert law, which predicts a linear change in absorbance with concentration. The RNA concentration range was within the manufacturer specifications for the NanoDrop (Thermo Fisher Scientific, Waltham, MA, USA). UV absorbance measurements were acquired using 1 µL of RNA sample in an ND-1000 spectrophotometer under the RNA-40 settings at room temperature (RT). Using this equation, an A260 reading of 1.0 is equivalent to ~40 µg/mL single-stranded RNA. The A260/A280 ratio was used to define RNA purity. An A260/A280 ratio of 1.8/2.1 is indicative of highly purified RNA. RNA extracts were directly amplified without reverse transcription to control the genomic DNA contamination. The RNAs were stored at −80° until use. 

### 4.3. Reverse Transcription

Four hundred nanograms of total RNA was reverse-transcribed with 2 μL of buffer 10X, 4.8 μL of MgCl2 25 mM, 2 μL ImpromII (Promega), 1 μL of RNase inhibitor 20U/l, 0.4 μL random hexamers 250 μM (Promega), 2 μL mix dNTPs 100 mM (Promega), and dd-water in a final volume of 20 μL. The reaction mix was carried out in a GeneAmp PCR system 9700 Thermal Cycle (Applied Biosystems, Foster City, CA, USA) under the following conditions: 5 min at 25 °C, 60 min at 42 °C, and 15 min at 70 °C for the inactivation of enzyme. The cDNAs were stored at −20° until use. 

### 4.4. Transcription Levels of IFNs, ISGs, TRIM28, SETDB1, pol Genes of HERV-H, -K, and -W, and env Genes of SYN1, SYN2, and MSRV by Real-Time PCR Assays

GAPDH was chosen as a reference gene in all determinations, being one of the most stable amongst reference genes and already used in our previous studies [45,51,56]. The relative quantification of mRNA concentrations of IFN-I, -II and -III signatures, TRIM28, SETDB1, HERV-H-pol, HERV-K-pol, HERV-W-pol, SYN1-env, SYN2-env, and MSRV-env was achieved using the ABI PRISM 7500 real time system (Thermofisher Scientific, Waltham, MA, USA).

Forty ng of cDNA were amplified using IFN-I and IFN-II signature mRNA expression kit BM-005, and BM-013, respectively (BioMole, Turin, Italy), in a 20 μL total volume reaction. The BM-005 kit evaluates six IFN-I ISGs: IFI44L, ISG15, IFIT1, RSAD2, SIGLEC, and IFI27 [118]. The BM-013 kit evaluates: IFN-γ CXCL9, CXCL10, and IDO1.

Expression levels of IFN-λ1, IFN-λ2, and IFN-λ3 were evaluated using 40 ng of cDNA in a 20 μL of total volume reaction containing 2.5 U goTaQ MaterMix (Promega), 1.25 mmol/L MgCl2, 500 nmol of specific primers, and 200 nmol of specific probes. The IFN-λ1 primers were: IFNL1F 5′-GAGGCATCTGTCACCTTC-3′, IFNL1R 5′-GGTTGACGTTCTCAGACA-3′, and the probe was IFNL1P 6FAM-5′-ACCTCTTCCGCCTCCTCACG-3′ (this study). The IFN-λ2 primers were: IFNL2F 5′- GCCACATAGCCCAGTTCAAG-3′, IFNL2R 5′- TCCTTCAGCAGAAGCGACTC -3′ [119] and the probe was IFNL2P 6FAM-5′- CTGTCTCCACAGGAGCTGCAGGCC -3′ (this study). The IFN-λ3 primers were: IFNL3F 5′- TCACCTTCAACCTCTTCC-3′, IFNL3R 5′-GAAGGGTCAGACACACAG-3′, and the probe was IFNL3P 6FAM-5′- TGGCAACACAATTCAGGTCTCG-3′ (this study). The probes were designed by Primer ExpressTM software version 3.0 (Thermofisher Scientific).

For TRIM28 and SETB1 expressions, 40 ng of cDNA were amplified using mRNA expression kits PP-044 [51] and PP-045, respectively (BioMole), in a 20 μL total volume reaction.

For HERV-H, -K, –W mRNA expressions 40 ng of cDNA were amplified using kits PP-054, -055, and -056, respectively, (BioMole) [51] in a 20 μL total volume reaction. The PP-BioMole-055 was derived from Schaban et al. [120]. The SYN1-env, SYN2-env, and MSRV-env mRNA expressions were also quantified by real-time PCR. Forty ng cDNA were amplified in a 20 μL of total volume reaction containing 2.5 U goTaQ MaterMix (Promega), 1.25 mmol/l MgCl2, 500 nmol of specific primers, and 200 nmol of specific probes. The SYN1 primers were: Sinc1F 5′-ACTTTGTCTCTTCCAGAATCG-3′, Sinc1R 5′-GCGGTAGATCTTAGTCTTGG-3′, and the probe was: Sinc1P 6FAM-TGCATCTTGGGCTCCAT-TAMRA [121]. The SYN2 primers were: Sinc2F-GCCTGCAAATAGTCTTCTTT-3′, Sinc2R- ATAGGGGCTATTCCCATTAG-3′ (Soygur and Sati 2016), and the probe was: Sinc2P-6FAM- TGATATCCGCCAGAAACCTCCC-TAMRA (this study). The MSRV primers were: MSRVF 5′-CTTCCAGAATTGAAGCTGTAAAGC-3′, MSRVR 5′-GGGTTGTGCAGTTGAGATTTCC-3′, and the probe was: MSRVP 6FAM-TTCTTCAAATGGAGCCCCAGATGCAG-3′-TAMRA [121]. The probes were designed by Primer ExpressTM software version 3.0 (Applied Biosystems, Foster City, CA, USA). 

The amplifications were run in a 96-well plate at 95 °C for 10 min, followed by 45 cycles at 95 °C for 15 s and at 60 °C for 1 min. Each sample was run in triplicate. Relative quantification of target gene transcripts was performed with the ΔΔCt method. Hence, fold change was calculated and results were expressed in corresponding arbitrary units, called relative quantification (RQ). Since we measured Ct for every target in all samples, we argue that our methods were suitable for HERV detection and quantification.

All analyses were performed in a laboratory of biosafety level 2 (BSL-2) according to the NIH [122] and WHO [123] guidelines.

### 4.5. Statistical Analysis

A one-way ANOVA test was used to compare the transcriptional levels of IFNs and ISGs, TRIM28 and SETDB1, HERV-H-pol, HERV-K-pol, HERV-W-pol, SYN1-env, SYN2-env, and MSRV-env between the four groups of children. The Mann–Whitney test was used to compare the transcriptional levels of each IFN and ISG, TRIM28, SETDB1, HERV-H-pol, HERV-K-pol, HERV-W-pol, SYN1-env, SYN2-env, and MSRV-env between each group of children with each other. The Spearman correlation test was used to evaluate the correlations between mRNA concentrations of IFNs and ISGs vs. those of TRIM28 and SETDB1, as well as between single HERV sequences vs. TRIM28 or SETDB1. Statistical analyses were done using the Prism software (GraphPad Software, La Jolla, CA, USA). In all analyses, *p* < 0.05 was taken to be statistically significant.

## Figures and Tables

**Table 1:** Demographics and clinical characteristics of SARS-CoV-2-infected children at the time of sampling. Group A: children with mild symptoms. Group B: children with severe symptoms. Group C: children with MIS-C. *n*: number; IQR: interquartile range, expressed as 25° and 75° quartile values; yrs: years; SD: standard deviation. CRP: C-reactive protein. Values upper * or below ** normal limit according to age-related cutoffs.

	Total (*n* = 64)	Group A (*n* = 36)	Group B (*n* = 17)	Group C (*n* = 11)
Median age (IQR)	7.8 yrs (2.3; 12.5)	8.0 yrs (1.3; 11.7)	10.1 yrs (3.8; 13.8)	6.8 yrs (4.7; 8.7)
Males (%)	37 (58.7)	25 (69.4)	7 (41.2)	5 (45.5)
Comorbidities, *n* (%)	27 (42.9)	16 (44.4)	10 (58.8)	1 (9.9)
Mean interval (+SD) from symptom onset and sampling	6.2 days (5.5)	5.8 days (5.6)	5.9 days (2.9)	7.6 days (3.3)
CRP > 10 mg/L, *n* (%)	18 (28.6)	3 (8.3)	7 (41.2)	8 (72.7)
Leucocytosis, *n* (%) *	6 (9.5)	3 (8.3)	1 (5.9)	2 (18.2)
Lymphopenia, *n* (%) **	16 (25.4)	4 (11.1)	7 (41.2)	5 (45.5)
Steroid treatment, *n* (%)	23 (35.9)	1 (2.8)	11 (64.7)	11 (100)

## Supplementary Materials

The following are available online at https://www.mdpi.com/article/10.3390/ijms22147481/s1.

## Abbreviations

COVID-19	Coronavirus disease 2019
HERVs	Human endogenous retroviruses
KRAB-ZFPs	Krüppel-associated box domain zinc finger proteins
IFN	Interferon
IFN-I	Type I interferon
IFN-II	Type II interferon
IFN-III	Type III interferon
MIS-C	Multisystem inflammatory syndrome in children
MSRV	Multiple sclerosis-associated retrovirus
PRR	Pattern recognition receptor
SAMHD1	Sterile alpha motif and HD-domain–containing protein 1
SARS-CoV-2	Severe acute respiratory syndrome coronavirus 2
SETDB1	SET domain bifurcated histone lysine methyltrasferase 1
SYN1	Syncytin 1
SYN2	Syncytin 2
TRIM28	Tripartite motif containing 28
