# Supplementary figures and images for: COVID-19 in Children: Expressions of Type I/II/III Interferons, TRIM28, SETDB1, and Endogenous Retroviruses in Mild and Severe Cases
Source: Int J Mol Sci. 2021 Jul 13;22(14):7481. doi: 10.3390/ijms22147481 (PMC8303145; doi:10.3390/ijms22147481)

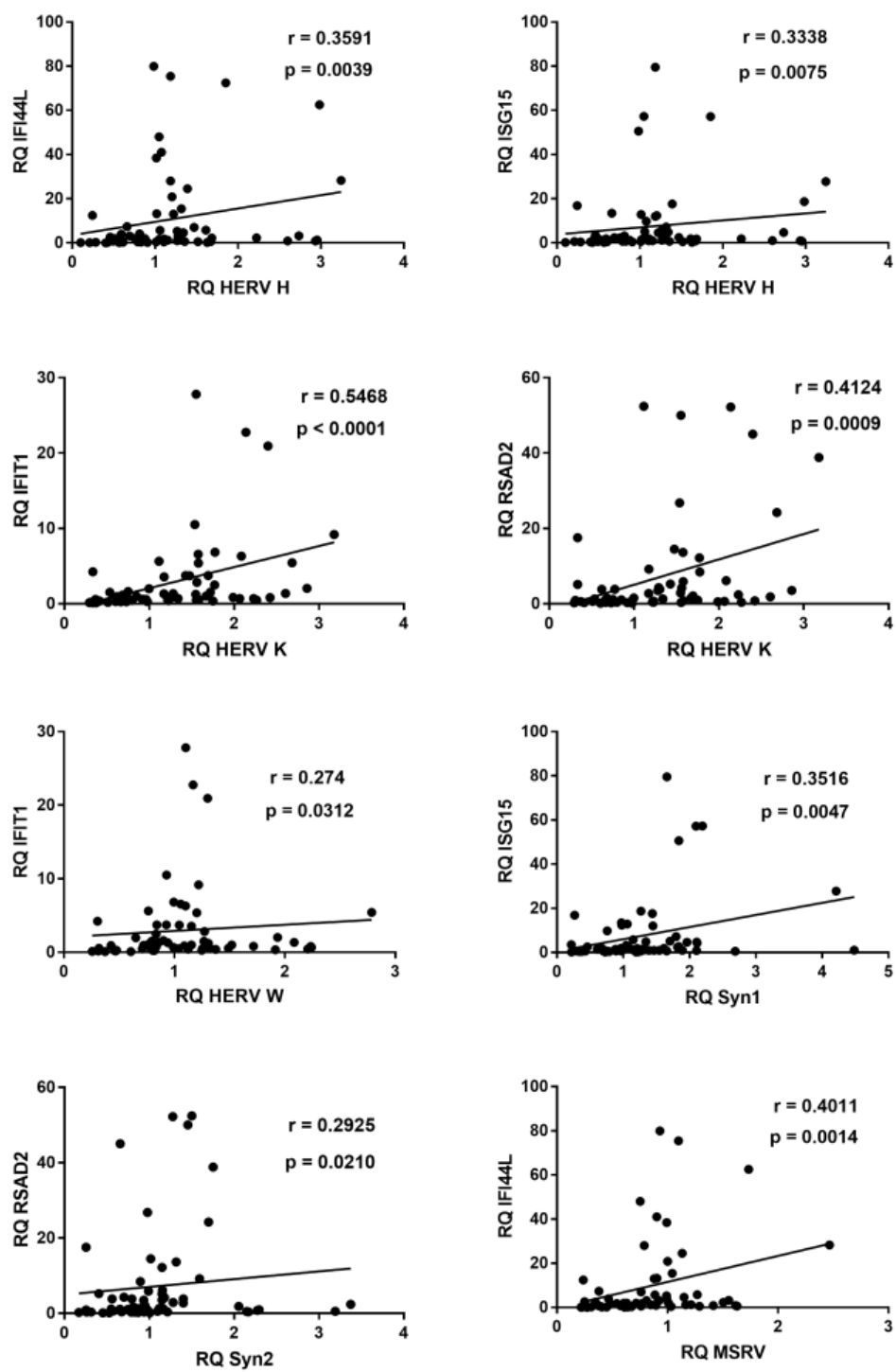

Figure S1

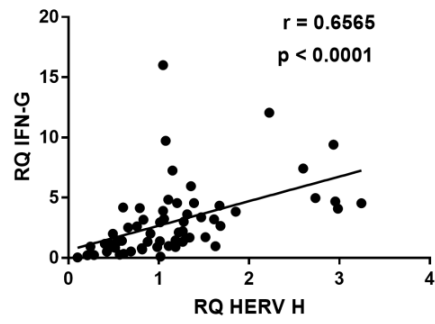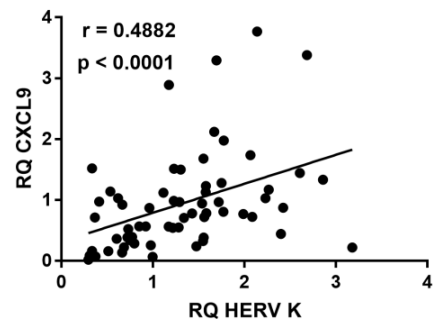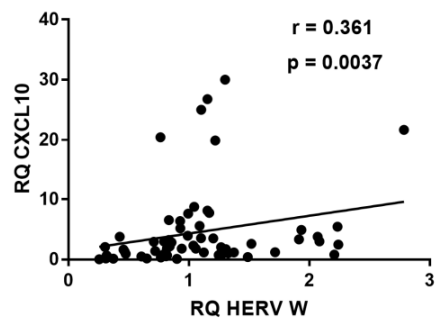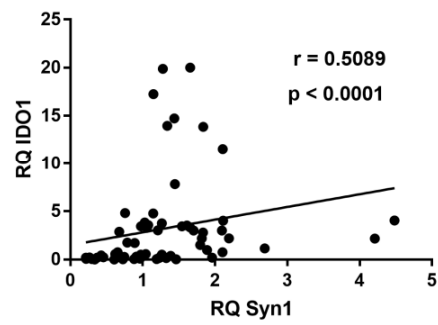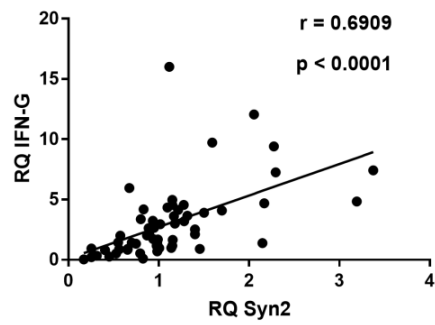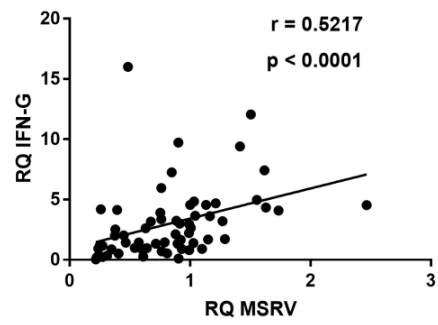

Figure S2

Supplement: Supplementary file 1 [file ijms-22-07481-s001.zip › ijms-1258528-Supplementary Materials.pdf]
